# Human β-D-3 Exacerbates MDA5 but Suppresses TLR3 Responses to the Viral Molecular Pattern Mimic Polyinosinic:Polycytidylic Acid

**DOI:** 10.1371/journal.pgen.1005673

**Published:** 2015-12-08

**Authors:** Fiona Semple, Heather MacPherson, Sheila Webb, Fiona Kilanowski, Laura Lettice, Sarah L. McGlasson, Ann P. Wheeler, Valerie Chen, Glenn L. Millhauser, Lauren Melrose, Donald J. Davidson, Julia R. Dorin

**Affiliations:** 1 MRC Centre for Inflammation Research, University of Edinburgh, Queen’s Medical Research Institute (QMRI), Edinburgh, United Kingdom; 2 MRC Human Genetics Unit, Institute of Genetics and Molecular Medicine (IGMM), University of Edinburgh, Edinburgh, United Kingdom; 3 Department of Chemistry and Biochemistry, University of California, Santa Cruz, Santa Cruz, California, United States of America; Ospedale San Pietro Fatebenefratelli, ITALY

## Abstract

Human β-defensin 3 (hBD3) is a cationic host defence peptide and is part of the innate immune response. HBD3 is present on a highly copy number variable block of six β-defensin genes, and increased copy number is associated with the autoimmune disease psoriasis. It is not known how this increase influences disease development, but psoriasis is a T cell-mediated disease and activation of the innate immune system is required for the initial trigger that leads to the amplification stage. We investigated the effect of hBD3 on the response of primary macrophages to various TLR agonists. HBD3 exacerbated the production of type I Interferon-β in response to the viral ligand mimic polyinosinic:polycytidylic acid (polyI:C) in both human and mouse primary cells, although production of the chemokine CXCL10 was suppressed. Compared to polyI:C alone, mice injected with both hBD3 peptide and polyI:C also showed an enhanced increase in Interferon-β. Mice expressing a transgene encoding hBD3 had elevated basal levels of Interferon-β, and challenge with polyI:C further increased this response. HBD3 peptide increased uptake of polyI:C by macrophages, however the cellular response and localisation of polyI:C in cells treated contemporaneously with hBD3 or cationic liposome differed. Immunohistochemistry showed that hBD3 and polyI:C do not co-localise, but in the presence of hBD3 less polyI:C localises to the early endosome. Using bone marrow derived macrophages from knockout mice we demonstrate that hBD3 suppresses the polyI:C-induced TLR3 response mediated by TICAM1 (TRIF), while exacerbating the cytoplasmic response through MDA5 (IFIH1) and MAVS (IPS1/CARDIF). Thus, hBD3, a highly copy number variable gene in human, influences cellular responses to the viral mimic polyI:C implying that copy number may have a significant phenotypic effect on the response to viral infection and development of autoimmunity in humans.

## Author Summary

Defensins are classically known as antimicrobial peptides due to their ability to rapidly kill pathogens including bacteria, viruses and fungi. They are produced in the presence of infectious agents at body surfaces exposed to the environment. Increasingly, their functional repertoire is expanding, and they have been shown to modulate the immune system. In humans, there is a block of six β-defensin genes that varies in copy number in the population. Individuals with an increased number of β-defensin genes have an increased likelihood of developing the skin autoimmune disease psoriasis. It is not known how this increase in gene copy number influences development of the disease, and psoriasis is a complex interplay of genomic and environmental factors that trigger disease progression and include exposure to viruses. We examined whether a molecular pattern characteristic of viruses produces an altered immune response in the presence of the defensin human β-defensin 3 (hBD3). We find that hBD3 triggers a larger interferon defence response to this viral mimic by increasing accessibility to a cellular receptor that recognises viral patterns. Interferon is known to be important in autoimmunity and our work may explain why individuals with increased β-defensin number are predisposed to develop psoriasis.

## Introduction

HBD3 is a member of the β-defensin multigene family. The amphipathic, antiparallel β-sheet structure, stabilised by disulfide bonds, via six canonical cysteines is conserved throughout evolution [1] and between family members despite significant sequence diversity [2]. These powerful cationic antimicrobials directly kill fungi, bacteria and viruses, and recently it has become clear that this gene family has roles in other processes including male fertility, immunomodulation and inflammatory disease [3]. Defensins are primarily expressed from mucosal surfaces, some exclusively in the reproductive tract and others in skin, intestine and gingival surfaces [4–6]. Of β-defensin genes, hBD3 is probably the most versatile and studies both *in vitro* and *in vivo* demonstrate its ability to chemoattractant immune cells [7]; encourage wound healing [8] and modulate innate signalling [9–11]. HBD3 (gene name *DEFB103*) and its mouse orthologue (Defb14) are promiscuous ligands with ability to bind the receptors CCR6, CCR2, CXCR4. In addition, a dominant mutation in the *DEFB103* gene in dogs and wolves causes an increase in canine β-defensin 3 (CBD103) peptide level allowing off-target binding to melanocortin receptor 1 (MC1R), which results in black coat colour [7, 12–15].

*DEFB103* is present on hypervariable clusters of six β-defensin genes and alteration in copy number may influence innate immune responses. Increased copy number of the cluster is associated with psoriasis [16, 17]. Increased defensin peptide level has been reported in serum of psoriasis patients, although the influence of defensins on the pathogenesis of the disease is not understood. Psoriasis is a T cell-mediated disease predominantly orchestrated by Th-17 cells. Amplification of the disease process is triggered by an initial phase modulated by an increase in innate immune signalling through pattern recognition receptors (PRR) such as toll like receptors (TLR) [18]. Psoriatic patients have an increase in dendritic cells and cationic antimicrobial peptides in the skin [19]. It has been shown that self and viral nucleic acids trigger an increase in the type I Interferon-α response of plasmacytoid dendritic cells (pDC) that are specialised cells for Interferon-α production through TLR9[20]. Blocking production of Interferon-α by these cells prevents T cell–dependent development of psoriasis in a xenograft model. The antimicrobial peptide LL-37 has been identified as a molecule that encourages recognition of self DNA and RNA through TLRs on pDC to induce release of Type I Interferon [21]. Recently antimicrobial peptides hBD2, hBD3 and lysozyme have also been shown to bind self-DNA and activate pDC through TLR9 to release Interferon-α. The presence of these peptides in psoriatic plaques suggests a concerted role for them in the pathogenesis of psoriasis [9, 19].

Here we determine the effect of hBD3 on the response of primary macrophages to known pathogen-associated molecular patterns (PAMPs) to investigate the influence of hBD3 on signalling from innate immune receptors. We have previously shown that hBD3 suppresses the TLR4-mediated response to bacterial lipopolysaccharide (LPS) which is mediated by both MyD88(Myeloid Differentiation Primary Response 88) and TICAM1 (Toll-Like Receptor Adaptor Molecule 1-also known as TRIF) [10]. We show that polyI:C in the presence of hBD3 has an exacerbated Interferon-β response and decreases CXCL10 production, *in vitro* and *in vivo* in both mice and human primary cells. PolyI:C is a synthetic double stranded RNA (dsRNA) and consequently a viral ligand mimic, which is recognised by endosomally located TLR3 and also by cytoplasmic RIG-I-like receptors (RLRs) [22]. Recently high molecular weight (HMW) or long poly:IC has been shown to preferentially access RLRs in conventional dendritic cells [23]. The RLRs include RIG-I (also known as DDX58 (DEAD (Asp-Glu-Ala-Asp) box polypeptide 58) and MDA5 (Melanoma Differentiation-Associated protein 5, also known as IFIH1 -interferon induced with helicase C domain 1) [24]. HMW polyI:C is recognised primarily by MDA5 (without the need for transfection) and secondarily (with transfection) by RIGI [23]. Activation of MDA5 and consequent interferon-β production has been shown to be associated with autoimmune disorders [25–28]. Both TLR3 and RLR types of receptor are MYD88 independent, with MDA5 and RIGI requiring the recruitment of the adaptor protein MAVS (mitochondrial antiviral signalling protein -also known as VISA, IPS-1 and CARDIF). Signalling through the MAVS pathway in macrophages results in IRF3/7 driven expression of type I Interferon and NF-ᴋB induction of inflammatory cytokines and chemokines [29, 30]. TLR3 specifically associates with the adaptor TICAM1 and mediates signal transduction which activates IRF3 and NF-ᴋB [30]. Our studies have been carried out using HMW polyI:C throughout. We dissect the effect on the pathways responsible for the altered response of macrophages to HMW polyI:C in the presence of hBD3 and reveal the mechanism that enables increased Interferon-β and decreased CXCL10 production.

## Results

### HBD3 exacerbates polyI:C induced signalling *in vitro* and *in vivo*

**Fig 1:**
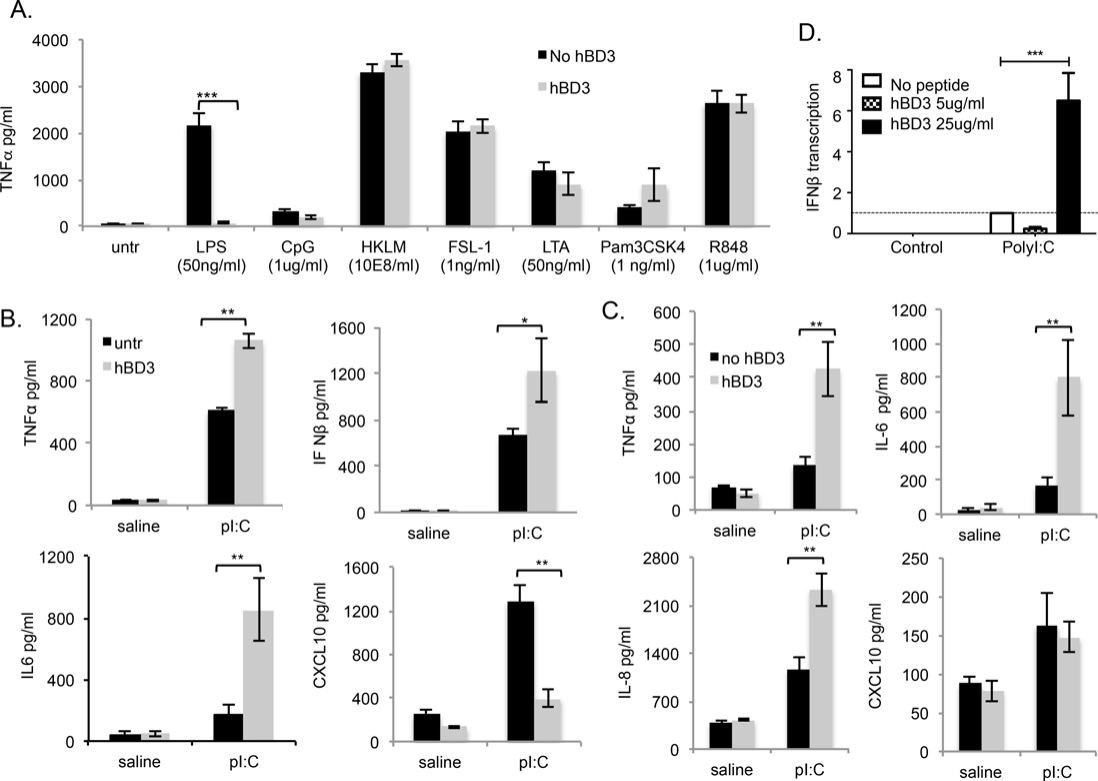
hBD3 exacerbates stimulation by the viral mimic HMW polyI:C but does not increase stimulation by other TLR ligands (A) BMDM from 3 separate wildtype (wt) mice were incubated overnight with HKLM (Heat Killed *Listeria monocytogenes*) (TLR2 ligand), FSL-1 (synthetic lipoprotein Pam2CGDPKHPKSF derived from *Mycoplasma salivarium) (TLR2/6 ligand)*, LTA (Lipoteichoic acid from *S. aureus* (TLR2 ligand), Pam3CSK4 (synthetic mimic of bacterial triacylated lipopeptide (TLR2/1 ligand), R848 (imidazoquinoline compound (TLR7 ligand), CpG (TLR9), TLR4 (LPS) or (B) HMW polyI:C (pI:C) (TLR3/RLR agonist) (10µg/ml) with and without hBD3. (C) PMA-differentiated THP-1 cells were treated with HMW polyI:C (10µg/ml) with or without 5 mg/ml hBD3. TNF-α, IL-6, IL-8 and CXCL10 induction were measured by ELISA *p≤0.05, **p≤0.01 student t-test (D) Human primary PBMDM were exposed to polyI:C as indicated, Interferon-β (IFNβ) gene expression was measured by qRT-PCR. ***p<0.001, 2-way ANOVA.

We previously reported that hBD3 suppresses Toll Like Receptor 4 (TLR4)-induced signalling in response to LPS both through MYD88 and TICAM1 pathways [10]. To investigate the effect of hDB3 on other TLR pathways we exposed primary bone marrow derived macrophages from mice (BMDM) to a variety of TLR ligands and found that the response to TLR 2, 2/6, 1/2, 7 or 9 ligands were not significantly altered in the presence of hBD3 (Fig 1A). In agreement with our previous findings, hBD3 inhibited LPS-induced TNFα. In contrast, the response to HMW polyI:C was significantly exacerbated in the presence of hBD3, increasing TNF-α; Interferon-β and IL-6 production, however hBD3 significantly suppressed the CXCL10 (also known as IP10) response to polyI:C (Fig 1B). The enhancing effect of hBD3 on polyI:C cytokine induction, was also seen in the human monocytic cell line THP1, where hBD3 significantly enhanced polyI:C-induced TNFα, IL-6 and IL-8. However, in contrast to what we observe in mouse cells, there was no significant effect of hBD3 on polyI:C-induced CXCL10 in human cells (Fig 1C). In addition this enhancing effect was also seen on Interferon-β gene expression in primary human peripheral blood monocyte derived macrophages (PBMDM), measured by qRT-PCR (Fig 1D).

**Fig 2:**
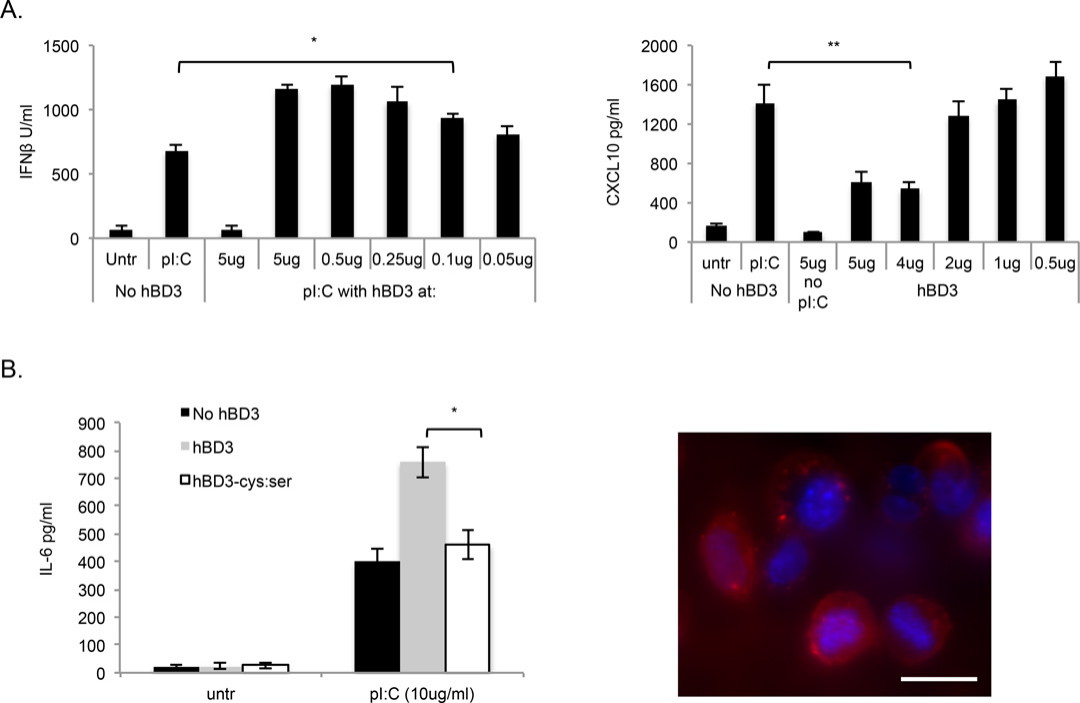
HBD3 effect on Interferon-β response to polyI:C is potent and is structure dependent. (A) BMDM from 3 separate mice were treated for 18hr with pI:C (10μg/ml) in the presence and absence of reducing concentration of hBD3 or (B) modified hBD3-cys:ser as indicated and IFNβ, CXCL10 and TNFα measured by ELISA. Image in B shows BMDM treated with 0.5μg/ml TAMRA-hBD3-cys:ser (10 min) observed by fluorescent microscopy. Scale bar 10μM.

The amount of hBD3 required to induce an enhanced Interferon-β response to polyI:C differed from that required to decreased CXCL10 response (Fig 2A). PolyI:C-induced CXCL10 was inhibited by 4μg/ml hBD3, but no longer inhibited at 2μg/ml hBD3, whereas concentrations as low as 0.05 μg/ml hBD3 enhanced the Interferon-β response to polyI:C, although at this concentration the effect is beginning to diminish. We tested the importance of the hBD3 cysteine-stabilised structure using hBD3 with the canonical defensin motif of six cysteines (which form three intramolecular disulfide bonds) replaced with serines. This modified peptide (hBD3 Cys:Ser) did not augment cytokine production in response to polyI:C, suggesting that the enhancing effect of hBD3 on polyI:C signalling is dependent on the 3-dimensional structure of the hBD3 peptide (Fig 2B). This lack of effect was not due to the inability of the linear peptide to rapidly enter the cell as TAMRA(tetramethylrhodamine azide)-labelled hBD3 Cys:Ser peptide entered BMDM in 10 min (Fig 2B) similar to canonically folded hBD3, as shown in our previous studies[10].

**Fig 3:**
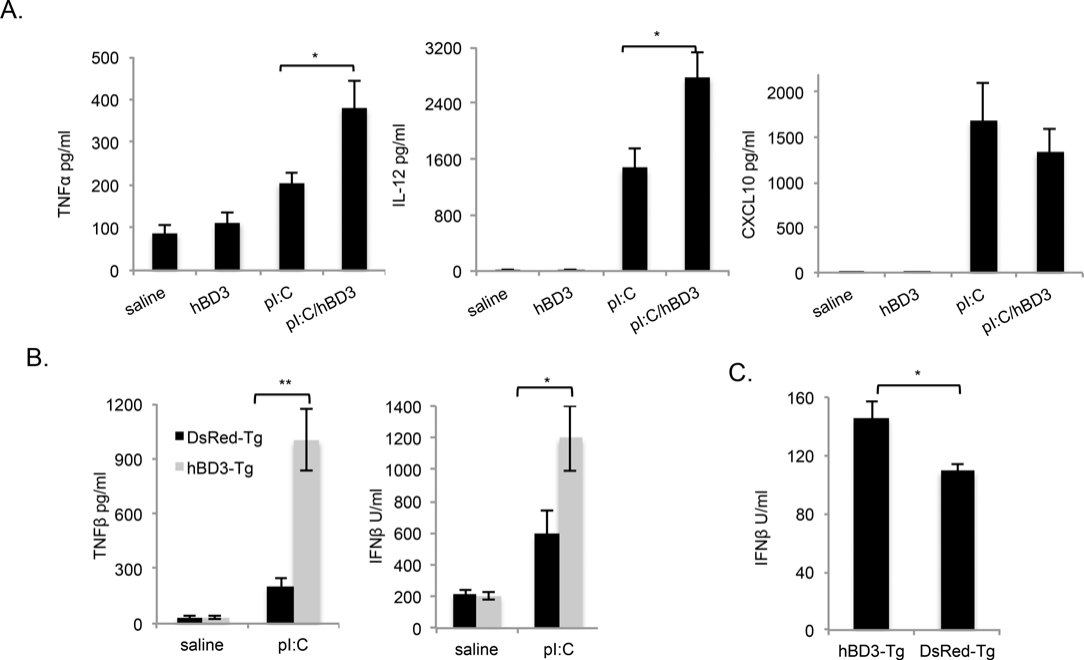
HBD3 enhances the effects of polyI:C *in vivo*. (A) Wild type (wt) mice were injected intraperitoneally (i.p.) with polyI:C (pI:C) (100μg/mouse) in the presence or absence of hBD3 (20μg/mouse) as indicated and cytokines measured by ELISA. (B) hBD3-Tg and DSRed-Tg control mice were injected i.p. with pI:C (100μg/mouse), after 4hr serum cytokine levels were measured by ELISA. (C) Wt and hBD3-Tg mice were bled and serum IFN-β measured by ELISA**p<0.01, *p<0.05 student t-test (n = 6 separate mice for each treatment group).

To test whether these *in vitro* effects were relevant *in vivo*, we injected wild type mice with polyI:C in the presence of synthetic hBD3 peptide (Fig 3A). We found that TNF-α and IL12p40 responses to polyI:C were significantly increased in the presence of synthetic hBD3. Interestingly the cytokine CXCL10 was not decreased in the presence of hBD3 (Fig 3A) although there was a trend for CXCL10 to be lower in the presence of hBD3- in keeping with the significant reduction of this cytokine seen in the BMDM stimulated with polyI:C and hBD3.

There is a concern that unless synthetic hBD3 is correctly oxidised and the correct disulphide bonding achieved, the properties of the peptide may be altered [7]. The importance of structure is confirmed in our experiment with hBD3 Cys:Ser shown in Fig 2B. The oxidised synthetic peptide we used here (obtained from Peptide Institute, Japan) gives details of preparation and oxidation [31] and implies correct cysteine bonding (C1-C5; C2-4 and C3-C6). However, in order to investigate our effect with hBD3 oxidised in vivo, we expressed the gene (*DEFB103*) that encodes hBD3, as a transgene in mice. Transgenic mice were made using the pCAAG promoter (as described previously by Candille et al. [14]) by introducing a transgene containing the genomic copy of *DEFB103* into ES cells. The vector (shown in S1A Fig) expresses hBD3 only after CRE recombinase-mediated deletion of the floxed *DsRed*, *puromycin* STOP spacer. After deletion, hBD3 and EGFP are expressed as a polycistronic mRNA and translated as independent proteins using an IRES site. We made several ES cell lines with the *DEFB103* expression construct and isolated a clone with strong expression by virtue of DsRed expression and a single site of insertion of the transgene (located to the sub-telomeric region of chromosome 12 by FISH and chromosome painting (S1B Fig). The control DsRed transgenic mice were made with this ES clone. HBD3 transgenic mice were made from the same ES clone after treatment with CRE recombinase. These cells had strong expression of EGFP and hBD3 and only the ES cells containing the CRE-excised vector showed expression of *DEFB103* mRNA (S1C Fig). HBD3 mRNA expression was detected in all tissues tested from mice made using the hBD3-Tg ES cells and hBD3 protein was detected in various tissues including BMDM by immunohistochemistry (S1D Fig) using an hBD3 monoclonal antibody [32]. No hBD3 was detected in DsRed-Tg control mice. The effect of polyI:C exposure in mice expressing physiologically secreted hBD3 was tested by exposing heterozygote hBD3-Tg and DsRed-Tg mice to polyI:C (100μg, i.p.). The transgenic animals expressing hBD3 demonstrated an increased level of both Interferon-β and TNF-α (Fig 3B). In addition, homozygous transgenic hBD3 mice had a significantly raised basal level of Interferon-β compared to controls (Fig 3C). A number of additional cytokines were investigated, including IL-6 and CXCL10. hBD3-Tg mice treated with polyI:C showed a trend towards enhanced IL-6 induction and reduced CXCL10 induction compared to controls, however these data did not reach significance (see S2 Fig).

### HBD3 and lipofectamine increase cellular uptake of polyI:C but induce diverse inflammatory responses

**Fig 4:**
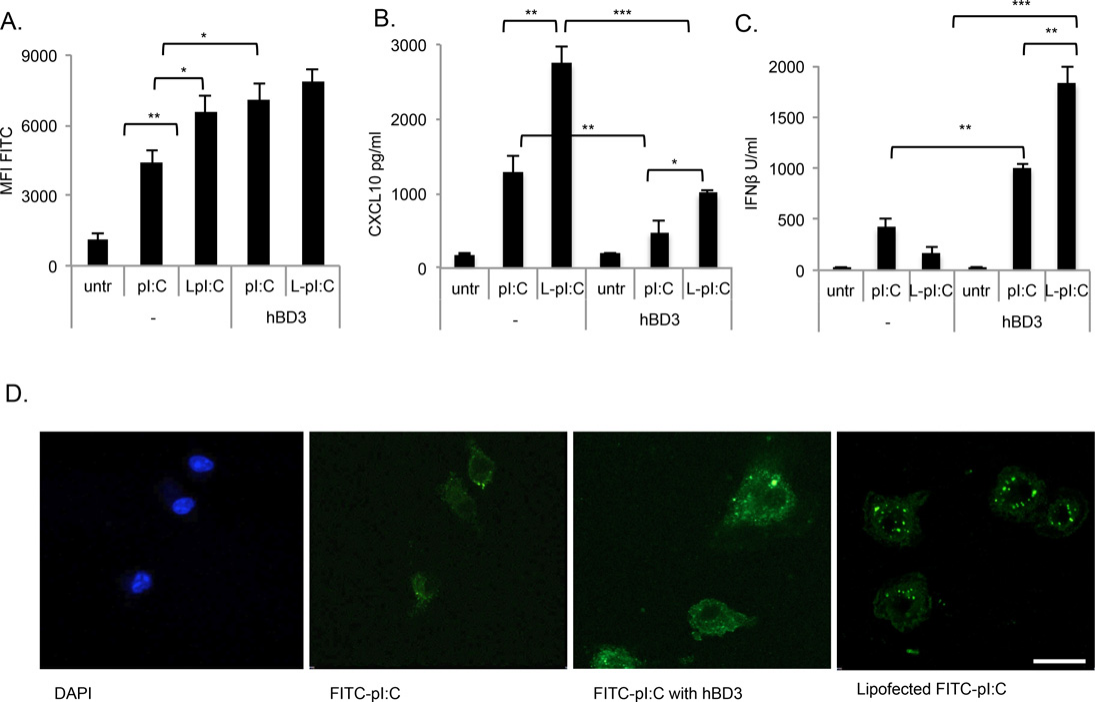
hBD3 does not mimic the effects of lipofectamine. BMDM from 3 separate wildtype mice were treated with pI:C, or pI:C pre-complexed with lipofectamine (at 1:100 dilution, 10μl/ml for 15min), in the presence or absence of hBD3. After 18hr cells were, (A) analysed by flow cytometry and culture supernatants were analysed by ELISA to measure (B) CXCL10 and (C) IFNβ ***p<0.001, **p<0.01, *p<0.05. (D) BMDM, grown on glass slides, were treated with 10mg/ml FITC-pI:C, without and with hBD3, without and with lipofectamine as labelled. Representative slides of each treatment visualised by confocal microscopy, are shown.

HMW polyI:C enters macrophages without a transfection reagent, however complexing with the cationic lipid, Lipofectamine 2000, enhances the amount of polyI:C entering cells by endocytosis, allowing more ligand to be available to both endosomal and cytoplasmic receptors [33]. We hypothesised that hBD3, being positively charged, may form complexes with polyI:C, enhancing uptake in a similar way to lipofection. To investigate this, we compared cellular uptake of FITC-labelled polyI:C in the presence of Lipofectamine 2000 and/or hBD3, by flow cytometry. Compared to polyI:C alone, the addition of hBD3 significantly increased the amount of labelled ligand per cell (Fig 4A). Lipofectamine alone also significantly augmented the amount of polyI:C entering cells, with the amount of FITC-polyI:C uptake in the presence of Lipofectamine not differing significantly from the level of FITC-polyI:C in hBD3 treated cells. Uptake in the presence of both lipofectamine and hBD3 was similar to either hBD3 or lipofectamine alone, so no additive effect was apparent.

We further compared the effects of lipofectamine and hBD3 on the production of Interferon-β and CXCL10 by polyI:C. Transfecting polyI:C with lipofectamine into wildtype BMDM resulted in enhanced CXCL10 production (Fig 4B). In contrast hBD3 inhibited CXCL10 induction by polyI:C. In the presence of a combination of lipofectamine and hBD3, CXCL10 production was still inhibited compared to polyI:C and lipofectamine, but to a lesser extent than hBD3 alone. In contrast, hBD3 and hBD3/lipofectamine significantly increased Interferon-β production in response to polyI:C in BMDM (Fig 4C). Lipofectamine and polyI:C did not demonstrate an enhanced Interferon-β response and lipofectamine decreased the amount of Interferon-β produced in response to polyI:C.

To visualise the uptake of polyI:C by BMDM we used FITC-labelled polyI:C (green fluorescence). Stronger fluorescence, was observed in cells when either hBD3 or lipofectamine was also present, supporting the flow cytometry data in Fig 4A showing both hBD3 and lipofectamine increase the entry of polyI:C into the cells. HBD3 increased the intensity of cyloplasmic polyI:C staining compared to either lipofectamine or polyI:C alone. In the presence of lipofectamine, polyI:C had a more punctate pattern within the cell, consistent with endosomal location. In the presence of hBD3, polyI:C appeared to have increased cytoplasmic distribution in addition to foci of staining. These results show that lipofectamine and hBD3, both enhance the amount of polyI:C that enters the cell but the different signalling responses triggered by each, suggests that hBD3 is directing polyI:C towards cellular compartments, that are not those targeted by the polyI:C-lipofectamine complexes.

### HBD3 enters the cell more rapidly than polyI:C—hBD3 and polyI:C do not co-localise

**Fig 5:**
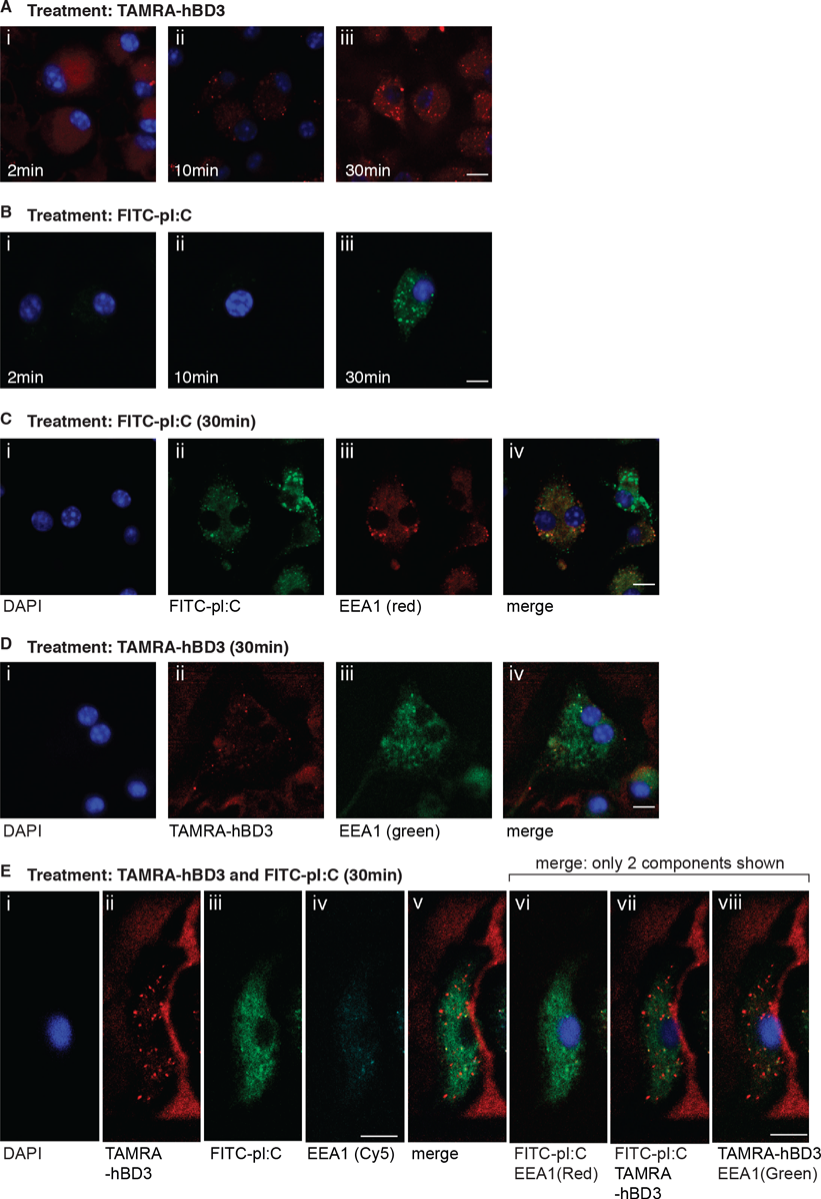
hBD3 enters cells more quickly than polyI:C and does not co-localise with polyI:C or endosome marker EEA1. Wildtype BMDM were treated with (A)TAMRA-hBD3 or (B) FITC-polyI:C (pI:C) and imaged at 2 (i),10 (ii) and 30 (iii) minutes. BMDM treated for 30 min with (C) FITC-pI:C (green) or (D) TAMRA-hBD3 were stained with anti-EEA1 antibody. DAPI (4',6-Diamidino-2-Phenylindole, Dihydrochloride) staining shown in C(i) and D(i); FITC-pI:C (green) and TAMRA-hBD3 (red) are shown in C(ii) and D(ii) respectively; EEA1 staining is shown in C(iii) and D(iii) (pseudocoloured red and green respectively); pI:C and hBD3 colocalisation with EEA1 are demonstrated C(iv) in and D(iv) merged images respectively, with Pearson co-efficients of r = 0.615(C(iv)) and r = 0.205(D(iv)). (E-i-viii) BMDM were treated (30 min) with FITC-pI:C and TAMRA-hBD3 and immunostained with EEA1-1 antibody. One representative cell from each slide is shown with filters allowing visualisation of single entities, E(i) DAPI staining for nucleus; E(ii) TAMRA-hBD3; E(iii) FITC-pI:C; E(iv) EEA1-1-Cy5 (blue), E(v) merge of Eii,iii and iv. E(vi)-(viii) are merged images showing only two components to assess co-localisation, (vi) polyI:C (green), EEA1-1 (pseudocoloured red) and hBD3 invisible; (vii) polyI:C (green) hBD3 (red) and EEA1-1 invisible; (viii) hBD3 (red) and EEA1-1(pseudocoloured green) and polyI:C invisible. Images are representative of all cells treated, and visualised by confocal microscopy. Additional single colour images of EEA1 pseudocolouring to complement 5E are shown in S3 Fig. Scale bars indicate 10 microns.

To further investigate the mechanism of the hBD3 effect on polyI:C we carried out fluorescence co-localisation studies using TAMRA-labelled hBD3, FITC-labelled polyI:C and immunohistochemistry for the early endosomal marker (EEA1). TAMRA-labelled hBD3 entered the cells by 10mins (Fig 5Aii), whereas FITC-polyI:C was not visible in the cells until 30 minutes (Fig 5Biii). FITC-polyI:C added to the cells without hBD3 (Fig 5B and 5C), demonstrated FITC fluorescence in punctate regions which stained to some extent with the early endosome marker EEA1-1 antibody (Pearson coefficient, r = 0.615; Fig 5Civ). HBD3 alone (Fig 5A and 5D) also localised to discrete regions however these did not co-localise with EEA1 staining (Pearson coefficient, r = 0.205; Fig 5Div). Adding TAMRA-hBD3 and FITC-polyI:C together onto BMDM, again showed more polyI:C entering the cell in the presence of hBD3 (Fig 5Biii, 5Cii vs 5Eiii). However we could see no evidence for co-localisation of hBD3 and polyI:C (Pearson coefficient, r = 0.073; Fig 5Evii). PolyI:C appeared more cytoplasmic in the presence of hBD3 than when it entered the cell alone and the co-localisation coefficient with EEA1-1 staining was lower than in the absence of hBD3. (Pearson coefficient, r = 0.562 compared to r = 0.615 Fig 5Civ versus Fig 5Evi). In the presence of polyI:C, hBD3 remained localised in the discrete foci similar to those seen with hBD3 treatment alone, and again hBD3 did not co-localise with EEA1 (Pearson coefficient r = 0.05; Fig 5Eviii). This implies that hBD3, alters the localisation of polyI:C allowing less polyI:C to access the early endosome. Although nucleic acids can induce type I Interferon by activation of TLR signalling [34] in the endosome, Interferon-β and IL-6 can also be produced by activation of cytoplasmic receptors.

### hBD3 exacerbates cytoplasmic MAVS-mediated signalling but suppresses TICAM1-mediated signalling

**Fig 6:**
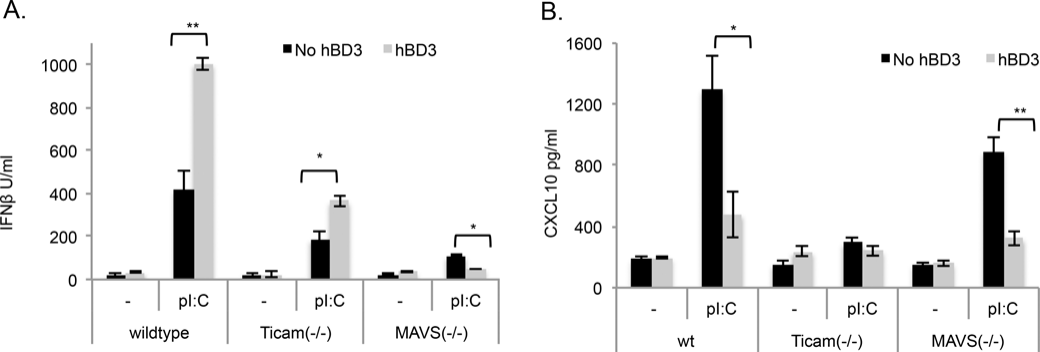
hBD3 inhibits TICAM1 signalling but enhances MAVS-dependent signalling. BMDM from wildtype, *Mavs(*-/-) or *Ticam1*(-/-) mice were treated with 10μg/ml pI:C in the presence or absence of hBD3. After 18hr cell culture supernatants were analysed by ELISA to measure (A) Interferon-β (IFNβ) and (B) CXCL10 **p<0.01, *p<0.05 (In each group BMDM were from 4 separate mice).

To examine the consequences of the altered localisation of polyI:C by hBD3 and to determine the signalling pathways responsible for production of Interferon-β and CXCL10, we used cells from knockout mice specific for the two main pathways known to be involved. Firstly, we determined the Interferon-β response of BMDM exposed to polyI:C in the absence of the TLR3 adaptor TICAM1 and found that this was reduced in *Ticam1-/-* cells compared to wild type BMDM, indicating that the majority of the polyI:C effect on Interferon-β was not TICAM1 dependent. In the presence of hBD3, the polyI:C-induced Interferon-β response in *Ticam1-/-* BMDM was still significantly enhanced compared to polyI:C alone (Fig 6A), which suggests that hBD3 is not enhancing signalling through the TLR3/TICAM1 pathway. Conversely, in the absence of MAVS, where Interferon production is only through TLR3/TICAM1 signalling, a smaller amount of Interferon-β was induced by polyI:C. In the presence of hBD3 this Interferon-β induction was significantly inhibited (Fig 6A), suggesting that hBD3 inhibits TLR3/TICAM1 signalling. Treatment of BMDM from *Ticam1*(-/-)*Mavs*(-/-) double knockouts with polyI:C did not induce Interferon-β indicating that all the Interferon-β response to polyI:C in BMDM is dependent only on these two pathways (S4A Fig).

In contrast to Interferon-β, the induction of CXCL10 by polyI:C in wildtype BMDM was significantly reduced in the presence of hBD3. In *Ticam1*(-/-) BMDM the response to polyI:C was eliminated (Fig 6B) demonstrating that production of this cytokine is controlled primarily by TLR3/TICAM1 activation. Supporting this finding, polyI:C-induced CXCL10 in *Mavs*(-/-) BMDM (a TLR3-TICAM1 dependent response) was not significantly different to wildtype cells indicating that MAVS does not influence CXCL10 production and the production of CXCL10 in response to polyI:C in *Mavs(-/-)* cells was still significantly inhibited by hBD3 (Fig 6B).

### hBD3 exacerbates MDA5 receptor signalling in response to polyI:C

**Fig 7:**
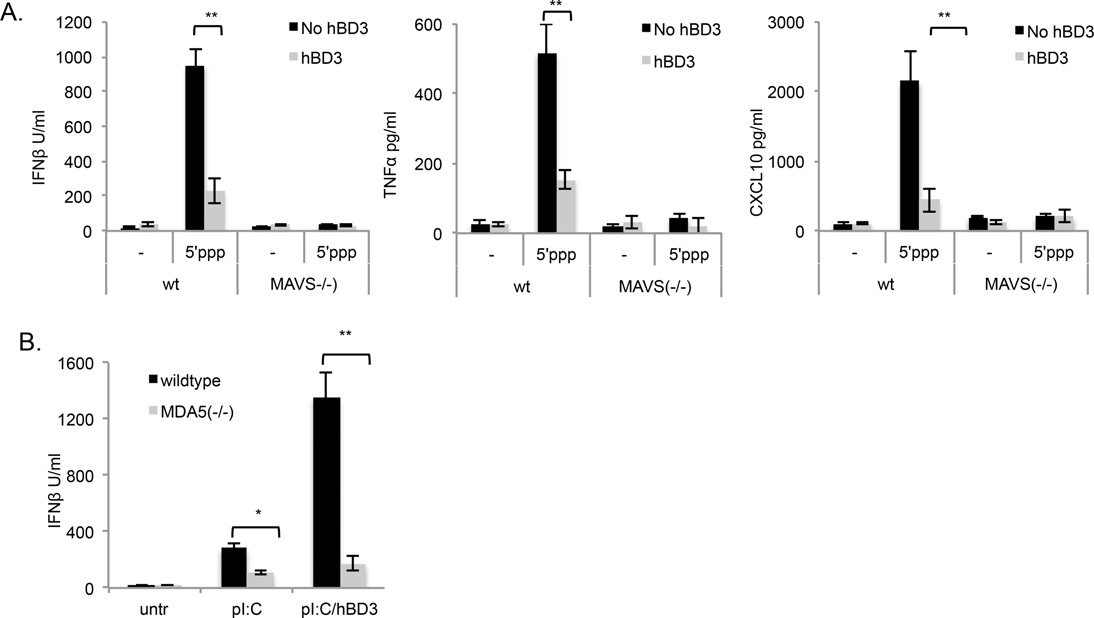
hBD3 inhibits RIGI signalling and enhances MDA5 signalling. (A) BMDM from wildtype and *Mavs*(-/-) mice were treated with 500ng/ml 5’ppp in the presence and absence of 5μg/ml hBD3 (B) wildtype and *Mda5* (-/-) BMDM were treated with pI:C in the presence and absence of hBD3. **p<0.01, *p<0.05, Student t-test. (In each group BMDM were from 4 separate mice).

Both RIGI and MDA5 are upstream of MAVS, so to dissect the effects of hBD3 on MAVS signalling we used the specific RIGI ligand, 5’ triphosphate double stranded RNA (5’ppp) and *Mda5(-/-)* mice [35]. Treatment of wildtype BMDM with 5’ppp resulted in a significant increase in Interferon-β, TNFα and CXCL10, and as expected with this ligand, these responses were dependent on the presence of MAVS (Fig 7A). The addition of hBD3 to the macrophages shortly after transfection of 5’ppp, resulted in a significant decrease in cytokine and Interferon-β production (Fig 7A), demonstrating that RIGI responses to 5’ppp are inhibited by the presence of hBD3. In contrast however, macrophages from *Mda5*(-/-) mice, revealed that Interferon-β induced by polyI:C was reduced compared to wildtype cells, indicating that MDA5 signalling was responsible for the majority of the polyI:C induced Interferon-β production (Fig 7B). This residual response which is likely to be TLR3-TICAM1 signalling, was not amplified in the presence of hBD3, indicating that MDA5 is required for the hBD3 enhancing effects on polyI:C-induced Interferon-β.

## Discussion

We show here that hBD3 enhances the production of various cytokines in response to polyI:C (TNF-α, IL6 in mouse cells and TNF-α, IL8 in human cells) and Interferon-β in both human and mouse cells. We demonstrate that this effect is dependent on the correct, disulfide stabilised structure of hBD3. Importantly, we show that this response is not specific to our synthetic hBD3 peptide, as transgenic animals expressing hBD3 from a genomic transgene, also demonstrate an increased type I Interferon response when injected with polyI:C. It was important to reproduce the results shown with our synthetic hBD3 peptide in this transgenic system to validate the augmenting effects of hBD3 as it has been demonstrated that synthetic peptides may be incorrectly folded giving misleading results [7]. The synthetic hBD3 peptide we use here, gives equivalent functional results to hBD3 produced *in vivo*.

When we dissect the pathways known to be activated by polyI:C, it is evident that although signalling through the RLR co-adaptor MAVS mediated pathway is up-regulated in the presence of hBD3, signalling through the endosomally located TLR3/TICAM1 pathway is suppressed. The inhibition of the TICAM1-mediated signalling pathway supports our findings with LPS, where we previously reported that hBD3-mediated inhibition of LPS signalling through TLR3/TICAM1 was lost in *Ticam1* KO mice and could be inhibited by hBD3 cDNA in HEK293 cells [10].

In our experiments here, we use HMW (long) polyI:C which activates MDA5 with or without transfection and show that in BMDM, MDA5 is predominantly responsible for the Interferon-β response. Interestingly, it has been demonstrated previously that LMW and HMW polyI:C are recognised by different receptors with HMW polyI:C being recognised by MDA5 and LMW polyI:C by RIGI [24]. In addition large RNA structures generated by viral replication are believed to be important in effectively triggering MDA5 [36]. Recently Zou *et al* reported that forced delivery of HMW polyI:C to the cytoplasm with transfection was not necessary for RLR stimulation in GM-DCs and CD11b^hi^CD24^lo^ DCs, although LMW polyI:C required transfection to interact with MDA5 [23]. We show here that similarly to the DCs, BMDM also take up HMW polyI:C effectively without transfection and activate MDA5. HBD3 exacerbates the signalling through MDA5. These researchers also report release of endosomal Cathepsin D and induction of necrosis by the activation of MDA5. We see no evidence of cell death (using an LDH assay, see S5 Fig) using polyI:C at 10μg/ml, but this is 5-fold less than that used by Zou et al [23].

**Fig 8:**
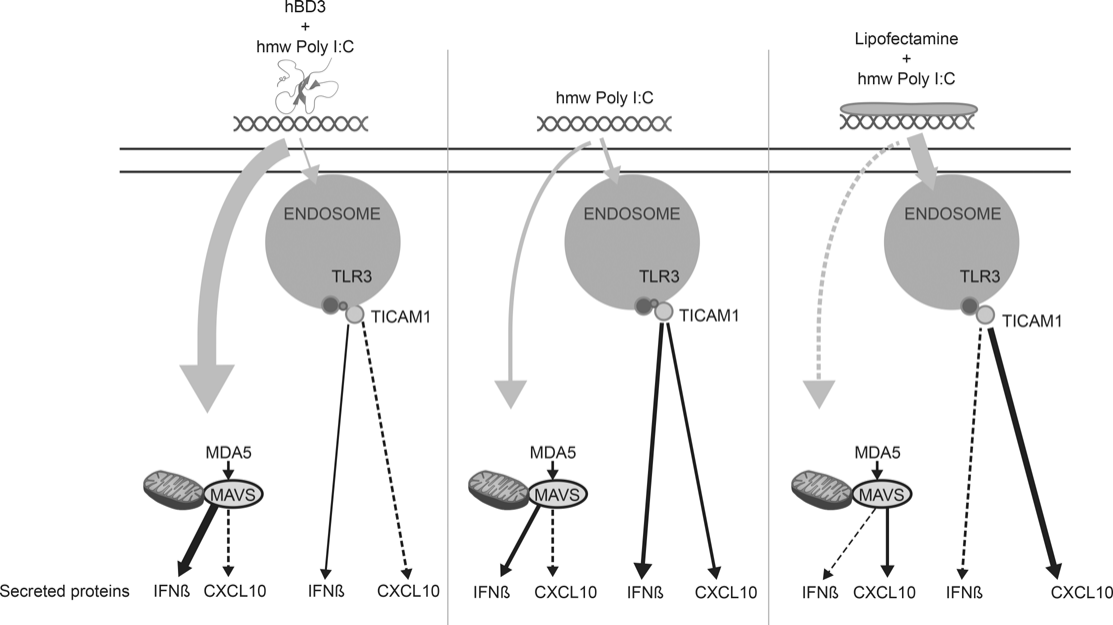
Schematic of hBD3 and Lipofectamine 2000 interaction with HMW polyI:C. Pale arrows indicate relative amounts of HMW polyI:C accessing different areas of the cell under our three different treatment conditions. The cell membrane is indicated by a double line, MAVS is located adjacent to the mitochondria. MDA5 is free in cytoplasm but goes to MAVS adaptor to generate the signalling cascade that results in IRF activation and production of Interferon-β (IFNβ). The black arrows indicate the strength of signalling that results in IFNβ or CXCL10 production with the dotted line indicating least signalling and the widest line indicating most signalling. Compared to polyI:C alone, hBD3 increases IFNβ production from MDA5 signalling and despite reducing the amount from TLR3, overall IFNβ levels rise. CXCL10 production from TLR3 is reduced by polyI:C in the presence of hBD3 because less is located at the endosome available for TLR3 signalling. In the presence of Lipofectamine, polyI:C production by TLR3/TICAM1 of CXCL10 increases due to increased endosomal signalling.

Cationic lipids such as Lipofectamine are known to cause endosomal localisation [33]. Although hBD3 is a cationic peptide we do not observe similar outcomes when we compare the effects of hBD3 with the actions of lipofectamine on polyI:C stimulation of macrophages. In wild type cells stimulated with polyI:C, hBD3 increased Interferon-β and decreased CXCL10 production. In contrast, polyI:C and lipofectamine increased CXCL10 production and decreased Interferon-β. This effect is likely to be due to the change in localisation of polyI:C as a result of being in the presence of lipofectamine or hBD3 (see Fig 8). Our immunostaining demonstrates that hBD3 encouraged polyI:C to be more cytoplasmic compared to lipofectamine which causes increased endosomal localisation. Despite the likely electrostatic interaction of polyI:C and the highly charged hBD3 (+11) in the cell, our cellular uptake experiments using fluorescently labelled derivatives revealed that at 30min after addition to the cells hBD3 and polyI:C do not co-localise appreciably. It is possible that initially they may have interacted, allowing polyI:C to access the cell as hBD3 has been described as having cell penetrating properties [37]. In the presence of hBD3, polyI:C does not localise to the early endosome so presumably the cytoplasmic location of the ligand allows an increase in interaction with MDA5. It is possible that cationic hBD3 complexed with polyI:C, enables the ligand to rapidly escape the acidic endolysosome, perhaps in a similar way to pH-dependent fusogenic peptides that assist macromolecules to access the cytoplasm [33]. However we see polyI:C localised to the early endosome (by EEA1 positive immunostaining) in the presence of hBD3 implying that the structure of the early endosome is not disrupted by the presence of hBD3.

The main consequence of increased MDA5 signalling in response to polyI:C is increased IFN-β. This increase is additive when lipofectamine is also present (S4B Fig). However no increase is observed in the absence of MAVS which implies that the exacerbated response requires the RLR. It may be that lipofectamine complexes the polyI:C to create higher order structures that activate MDA5 more optimally when [36] hBD3 increases its cytoplasmic localisation.

MDA5 is important in relation to autoimmunity and mutations that inactivate or reduce expression of MDA5 have been shown to protect individuals from type I diabetes mellitus risk [38, 39]. In addition, a mutant form of MDA5 in mice that is active without viral infection induces a type I Interferon-dependent autoimmunity with similarities to lupus [25]. However Interferon-β has also been described as a protector against some types of inflammation such as dextran sodium sulphate induced colitis [40–42] and this protection can be observed in mice that express increased Interferon-β in response to dsRNA-producing intestinal bacteria [40–42].

Increased copy number of the cluster of β-defensins on human chromosome 8 is linked to increased incidence of the autoimmune disease psoriasis and effective treatment of psoriasis with UV irradiation is linked to suppression of type I Interferon and Th17 cells [43]. In addition the most effective treatments currently for psoriasis are monoclonal antibodies directed against IL-17 cytokine production or IL-12p40 (the cytokine subunit common to both IL-12 and IL-23) [18, 44]. It is thus potentially highly significant that we see strong elevation of IL12-p40 subunit in mice injected with both hBD3 and polyI:C. It is also possible that the other defensins on the CNV cluster may also demonstrate this effect and we have shown that hBD2 also heightens the response of mouse BMDM to polyI:C (S6 Fig).

It has recently been shown that human pDC produce Interferon-α in response to self or other DNA through TLR9 [9, 19]. We show here that macrophages increase the Interferon-β response to polyI:C in the presence of hBD3 through MDA5. Production of type I Interferons is normally the consequence of pattern recognition receptors binding virally produced nucleic acid pathogen associated molecular patterns (PAMP), such as double stranded RNA (dsRNA) produced during viral replication. Psoriasis has been reported to be exacerbated by the use of Interferon-α as therapy for Hepatitis C [45] and by Interferon-β therapy for multiple sclerosis [46]. Investigation of the psoriasis transcriptome has identified an increase in RIG-I like receptors (RLR), which also recognise viral PAMP leading to type 1 Interferon production [47]. During a pathogen infection, hBD3 expression increases [32, 48], and hBD3 has been shown to demonstrate potent anti-viral action *in vitro* [49]. Expression in pDC, monocytes and epithelial cells of the non-copy number variable defensin hBD1 has been shown to increase in response to virus exposure, while expression of the murine orthologue of *DEFB103* (*Defb14*) increases in response to polyI:C [50, 51]. MDA5 is specialised for protecting mice against infection with various RNA viruses including picornaviruses (including Theiler’s and Mengo viruses and Encephalomyocarditis virus (EMCV)) as well as paramyxovirus and Norovirus [52, 53]. MDA5 knockout mice are highly susceptible to EMCV [35, 54]. During infection, rapid killing, detection and innate response are essential; therefore in this regard, high hBD3 copy number and potentiation of PRR may be beneficial. However an undesirable effect of increased copy number of the defensin cluster (and concomitant increase in expression of defensin peptides) may be over stimulation of PRRs leading to exuberant production of type I interferons. This double edged sword may provide protection against pathogens in the short term, but in the longer term contribute to the development of psoriasis in individuals with an increased copy number of the β-defensin cluster.

## Materials and Methods

### Animal and human ethical statements

Animal studies were covered by Project License (PPL 60/4475), granted by the UK Home Office under the Animal Scientific Procedures Act 1986, and locally approved by the University of Edinburgh Ethical Review Committee. Human venous blood was collected with written patient consent from healthy volunteers according to Lothian Research Ethics Committee approvals ♯08/S1103/38.

### Reagents

Ultra pure Lipopolysaccharide (LPS) from *E*. *coli* 0111:B4, Lipoteichoic acid *(*LTA), Pam_3_CSK_4_, FSL-1, HKLM, polyI:C (HMW), FlTC-labelled polyI:C (HMW), R848, CpG and 5’ triphosphate double stranded RNA (5’ ppp-dsRNA) were purchased from InvivoGen (San Diego, USA), M-CSF, ELISA DuoSets and IFNβ antibodies were obtained from R&D Systems, (Abington, UK). Fluorescently labelled secondary antibodies were purchased from Jackson ImmunoResearch Laboratories (PA, USA). hBD3 (GIINTLQKYYCRVRGGRCAVLSCLPKEEQIGKCSTRGRKCCRRKK) was from Peptides International, and cys-ser hBD3 and cys-ser-TAMRA hBD3 were from Almac (Almac Group Ltd, Craigavon, UK).

### TAMRA-hBD3 synthesis, purification, and folding

The peptide was produced on a CEM Liberty1 microwave peptide synthesizer using standard Fmoc (fluorenylmethyloxcarbonyl chloride) chemistry. Amino acids were purchased from AAPPTec and were assembled on H-Rink amide ChemMatrix resin. Fmoc protecting groups were removed using 20% piperidine and 0.1 M hydroxybenzotriazole (HOBt) in dimethylformamide (DMF). Amino acids were coupled using 5 molar equivalents of diisopropylcarbodiimide (DIC) and 10 molar equivalents of HOBt in DMF. The N-terminal labelling of peptides with fluorescent dye was performed on resin-bound peptide using 4 equivalents of 5-(and-6)-carboxytetramethylrhodamine, succinimidyl ester (5(6)-TAMRA SE purchased from Biotium) and 6 equivalents of diisopropylethylamine (DIEA) in DMF, incubating for 2 hours. The peptide resin was then rinsed with DMF to remove excess fluorescent dye, washed with dichloromethane (DCM), and dried. Cleavage of the peptide from resin was performed in a trifluoroacetic acid (TFA)/ triisopropylsilane (TIS)/ 1, 2-ethanedithiol (EDT)/ phenol (90:4:4:2) mixture for 90 min. The resin was filtered and the filtrate was added to 90 mL of cold dry diethyl ether. The precipitate was collected by centrifugation and the diethyl ether was discarded. The peptide was purified on a C18 reverse phase HPLC column and the correct molecular weight was confirmed by ESI-MS. Oxidative folding was achieved in folding buffer (0.5–1.0 M guanidine hydrochloride (GuHCl), 0.1 M Tris, 1 mM glutathione (GSH), 0.1 mM oxidized glutathione (GSSG), pH 8.5) at a peptide concentration of 0.1 mg/mL and stirred for 48 hours. Folding was monitored by reverse phase HPLC, which revealed one major species that was used in subsequent experiments. Folding procedures were developed to give the correct HBD3 structure, as verified previously by nuclear magnetic resonance structure determination (Nix et al., 2013). The folded products were purified on a C18 reverse phase HPLC column and identified as fully oxidized peptides by ESI-MS. Quantitative concentrations were determined with amino acid analysis at the molecular structure facility at UC Davis.

### Mouse strains

The *Mavs−/−* (*Cardif*, *Ips-1*, or *Visa*) mutant line was generated by the Tschopp group in Lausanne. It is homozygous-viable null mutant in the C57B6J background. Ticam1 (Trif) -/- mice and MDA5-/- mice were used with the generous permission of Professor Shizuo Akira (Osaka University, Japan)[35, 55]. hBD3-Tg and DsRed-Tg were constructed by electroporation of ScaI linearised parental vector, which has a 1.5 Kb genomic fragment of the entire hBD3 gene DEFB103 including exons 1 and 2 and the intervening intron cloned into pTLC plasmid (a kind gift from Josh Brinkman, Danish Stem Cell Centre, DanStem, University of Copenhagen) using DEFB103 primers with 5' NheI and PacI sites for cloning, into ES cells. Cells that strongly expressed DsRed were selected by FACS and used to make DsRed-Tg mice. Transient cre treatment of these cells produced DsRed negative cells due to lox -mediated excision of the DsRed gene and allowed expression of hBD3 and EGFP (see S1 Fig). The ES cells were made into macrophages using the method of (Yeung et al 2015) which were strong expressors of EGFP. Clones before and after CRE treatment were injected into blastocysts at the University of Edinburgh MRC Evans Building, Transgenic Unit.

### Cell-lines and primary cell preparation

THP-1 cells were grown in RPMI with 10% fetal bovine serum (FBS) and differentiated into macrophages by the addition of 150nM PMA, 2 days before treatment. Mouse primary macrophages (BMDM) were generated from femur bone marrow grown for 8 days in DMEM with 10% fetal bovine serum and 20ng/ml M-CSF. Cells, seeded at 2 x 10^5^ into 48 well plates, were grown without M-CSF for 24 hours prior to treatment. Replicate experiments were done with separate primary cell preparations from at least 3 mice for each experiment.

### Mouse BMDM treatment and ELISA

Mouse BMDM, seeded at 2 x 10^5^ cells into 48 well plates were treated in serum free media with TLR or RIGI agonists at the concentrations indicated in the figures, in the presence or absence of hBD3 (5μg/ml). After an 18 hour incubation at 37°C, 5% CO2, TNF-α, IL-6, CXCL10 and Interferon-β were measured using mouse DuoSet ELISA (R&D Systems). Statistical significance was determined by an unpaired t-test using GraphPad software, with values expressed as mean +/- SEM and p < 0.05 considered significant.

### Human PBMDM isolation, treatment and quantitative PCR

Human venous blood was collected from healthy volunteers according to Lothian Research Ethics Committee approval, using sodium citrate anticoagulant (Phoenix Pharma, Gloucester, UK), and cells were separated by Dextran sedimentation, followed by discontinuous, isotonic Percoll gradient centrifugation as previously described [56]. PBMC were incubated at 4×10^6^/mL in IMDM (PAA Laboratories, Somerset, UK) at 37°C, 5% CO_2_, for 1 h. Non-adherent cells were removed and adherent monocytes cultured for 6 days in IMDM with 10% autologous serum to generate monocyte-derived Mφ. PolyI:C was applied in concentrations indicated and cells harvested at 18 hours. RNA was isolated and human Interferon-β gene expression was measured using the Applied Biosystems Taqman Gene Expression Assay following the manufacturer’s instructions.

### Immunostaining

Cytospins of mouse BMDM were fixed in 4% PFA, washed, then blocked for 2 hours at room temperature (RT) in 10% donkey serum (Sigma, Poole, UK) in PBST (0.1% Tween in PBS). Slides were then incubated overnight at 4°C with hBD3 antibody (1:200) (DSHB, Iowa University, USA). After washing, slides were incubated with TxRd labelled anti-mouse antibody (1:400) for 2 hours at RT, further washed then mounted with Vectashield containing 1μg/ml DAPI. hBD3 immunostaining was visualised using a Zeiss Axioplan 2 microscope (Carl Zeiss UK Ltd., Welwyn Garden City, UK) equipped with Ludl filter wheel (Ludl Electronic Products Ltd, Hawthorne, NY, USA) and Chroma 83000 triple bandpass filter set (Chroma Technology Corp, Rockingham, VT, USA). In-house scripts written for IPLab (Scanalytics Corp, Fairfax VA, USA) were employed for image capture and image processing.

### Cellular localisation and confocal microscopy

BMDM (2 x 10^4^ cells/well) were cultured overnight on 8 well glass chamber slides (Nunc Inc, IL, USA) in DMEM with 10% FCS. Cells were treated with FITC-polyI:C (2.5μg/ml) in Optimem media (Life Technologies) with or without lipofectamine 2000 (Invitrogen, at 1:100 dilution, 10μl/ml) in the presence or absence of hBD3 or TAMRA-labelled HBD3 (0.5μg/ml). After 2, 10, 15 or 30 mins cells were washed in PBS and fixed in 4% PFA. For early endosome staining, cells were blocked with 10% donkey serum and incubated with anti-EEA11 antibody (Abcam, UK) for 1 hr at RT, then 30 min with donkey anti-rabbit Cy5. Cells were imaged using a 40x 1.3NA oil immersion objective on a Nikon A1R confocal microscope using Nikon Nis-Elements AR software for image acquisition (Nikon Instruments Europe, Netherlands). Image analysis was carried out in ImageJ (http://imagej.nih.gov/ij/). Pearsons coefficients were calculated using the JaCoP ImageJ plugin [57].

### PolyI:C delivery *in vivo*

Male C57 Black/6 mice (6–8 weeks old) and hBD3 transgenic male mice (8 weeks) were injected intraperinoneally (i.p.) with polyI:C (100μg/mouse) in 200μl of physiological saline. Half of the C57 Black/6 mice also received an i.p injection of synthetic hBD3 (20μg/mouse). After 4 hr, mice were killed by cervical dislocation, exsanguinated and serum levels of TNFα and Interferon-β measured by ELISA.

### Analysis of polyI:C uptake

Mouse BMDM plated at 1 x 10^6^ cells on a 6-well plate were treated with FITC-pI:C (10μg/ml) in the presence of lipofectamine 2000 (at 1:100 dilution, 10μl/ml) or hBD3 (5μg/ml). For treatment with polyI:C in the presence of lipofectamine 2000, media was replaced with optimem before the addition of polyI:C complexed with lipofectamine 2000 (L-pI:C) and addition of hBD3 was delayed for 5 min to avoid direct interaction with L-pI:C complexes. After 18 hours cells were gently washed and gently removed from the dish into PBS containing 1% BSA. Fluorescence was measured with a BD FACSARIAII SORP (BD Biosciences, Oxford, UK), using a 640nm laser (670/14nm bandpass filter). Data analysis was done using FlowJo Version 7.5.5 (Treestar Inc, Olten, Switzerland). This experiment was carried out on 3 different preparations of BMDMs

## Supporting Information

S1 Fig Creation of DsRed-Tg and EGFP hBD3-Tg mice. **A: Transgene vector diagram:** Control and hBD3 expressing vectors were created from pTLC vector (kind gift of Josh Brickman). CAG indicate the CAAG promoter (cytomeglavirus enhancer and chicken β-actin) and the arrow indicates direction of transcription; dsRed indicates the *DsRed* gene [58]; IRES indicates internal ribosome entry site; Puro indicates *puromycin* antibiotic resistance gene STOP is the translation termination signal and pA indicates polyadenylation signal; hBD3 is *DEFB103* genomic fragment with exons 1 and 2 indicated by ex1 and ex2; *EGFP* is Enhanced Green Fluorescent Protein. ES cells (E14(iv), that were 129/Ola derived), were transfected by electroporation with ScaI linearised vector as shown in the upper region of the vector S1A Fig. Cells expressing DsRed by FACS that were shown to have normal chromosome number of n = 40, were injected into 3.5d blastocysts from C57Bl/6J mice, to create chimaeric animals. The chimaeras were bred with C57Bl/6N mice to establish heterozygotes. Homozygous mice were established at 7 backcross generations by het x het crosses and homozygotes were identified by strong DsRed fluorescence evident using a hand held lamp with a light source excitation of 440–460 nm. Homozygotes were validated by breeding i.e. all offspring carried the transgene when mated with wildtype. The ES clone used to make DsRed-Tgs was treated transiently with *cre* recombinase by transfection of a *cre* expression plasmid (kind gift of Prof. Austin Smith). These cells now no longer expressed DsRed by FACS but were EGFP positive. **B: The site of transgene integration:** of the vector was identified by fluorescent in situ hybridisation (FISH) and shown by chromosome painting of targeted ES cells to be on chromosome 12. **C: hBD3 expression in ES cells with cre treated vector:** shows that following CRE treatment the ES cells express transcript from *DEFB103* (hBD3). Mw indicates Øx174 DNA cut with HaeIII and run on 3% agarose gel. DEFB103 RT-PCR from exon 1 to 2 produces a band of 250bp and this is only visible when cDNA from the ES cells following cre treatment are used. Mice made from these cells as described above, were named *hBD3-Tg* and appeared green when viewed with a fluorescent light source (excitation 510–540 nm) as heterozygotes. Homozygotes were derived after 7 backcross generations as for Ds-Red homozygotes described above. **D: hBD3-Tg mice with the DEFB103 transgene express hBD3.** BMDM from transgenic *DEFB103* expressing mice (hBD3Tg) were immunostained with an hBD3 monoclonal antibody (a and b) [32] [32]or not (b and d) and Texas red labelled second antibody and visualized by fluorescent microscopy: (a and c) control mice, (b and d) hBD3-Tg.
(PDF)

S2 Fig Effects of intraperitoneal polyI:C on hBD3-Tg mice. hBD3-Tg and DSRed-Tg control mice were injected i.p. with pI:C (100μg/mouse), after 4hr serum cytokine levels were measured by ELISA. (n = 6 separate mice for each treatment group). In pI:C treated hBD3-Tg mice there is a trend towards enhanced pI:C induction and reduced CXCL10 induction, compared to controls, however these data did not reach significance.
(PDF)

S3 Fig Confocal images to complement Fig 5E. Images showing EEA-1 pseudocoloured red or green as indicated and no other fluorescence revealed. Fig 5Eviii and 5Evi shown again for scale and indication of merged images with the pseudocoloured EEA-1. merged Scale bar 10 microns.
(PDF)

S4 Fig Double and single Mavs and Ticam1 knockouts additional data. Response of BMDM from (**A**) Mavs(-/-)/Ticam(-/-) double knockout mice, and (**B**) Mavs (-/-) or Ticam(-/-) single KO BMDM were treated for 18hr with polyI:C (10μg/ml) in the presence and absence of hBD3 (5μg/ml), with or without lipofectamine (L) as indicated (L-p:C is lipofectamine with polyIC). IFNβ, CXCL10 and TNFα in cell supernatants were measured by ELISA, *p<0.05, **p<0.01, ***p<0.005 student t-test.
(PDF)

S5 Fig PolyI:C with or without hBD3 does not cause cell death (LDH assay). BMDM from wt mice were treated for 18hr with polyI:C (pIC) (10μg/ml) in the presence and absence of hBD3 (5μg/ml). 5x10^4^ cells were exposed to each treatment in triplicate then NADH levels measured using an LDH-Cytotoxicity Colorimetric Assay (BioVision).
(PDF)

S6 Fig hBD2 also exacerbates the effect of polyI:C. BMDM from wild type mice were treated for 18hr with polyI:C (10μg/ml) in the presence and absence of hBD2 (5μg/ml). IFNβ in cell supernatants was measured by ELISA, *p<0.01, student t-test.
(PDF)
