# Correction: Human β-D-3 Exacerbates MDA5 but Suppresses TLR3 Responses to the Viral Molecular Pattern Mimic Polyinosinic:Polycytidylic Acid

**DOI:** 10.1371/journal.pgen.1005801

**Published:** 2016-01-08

**Authors:** Fiona Semple, Heather MacPherson, Sheila Webb, Fiona Kilanowski, Laura Lettice, Sarah L. McGlasson, Ann P. Wheeler, Valerie Chen, Glenn L. Millhauser, Lauren Melrose, Donald J. Davidson, Julia R. Dorin

There are errors in the phrase “β-D-3” in the article title. This should read “β-Defensin 3”. The correct title is: Human β-Defensin 3 Exacerbates MDA5 but Suppresses TLR3 Responses to the Viral Molecular Pattern Mimic Polyinosinic:Polycytidylic Acid. The correct citation is: Semple F, MacPherson H, Webb S, Kilanowski F, Lettice L, McGlasson SL, et al. (2015) Human β-Defensin 3 Exacerbates MDA5 but Suppresses TLR3 Responses to the Viral Molecular Pattern Mimic Polyinosinic:Polycytidylic Acid. PLoS Genet 11(12): e1005673. doi:10.1371/journal.pgen.1005673.
