# Supplementary figures and images for: Human β-D-3 Exacerbates MDA5 but Suppresses TLR3 Responses to the Viral Molecular Pattern Mimic Polyinosinic:Polycytidylic Acid
Source: PLoS Genet. 2015 Dec 8;11(12):e1005673. doi: 10.1371/journal.pgen.1005673 (PMC4672878; doi:10.1371/journal.pgen.1005673)

Figure S1A:

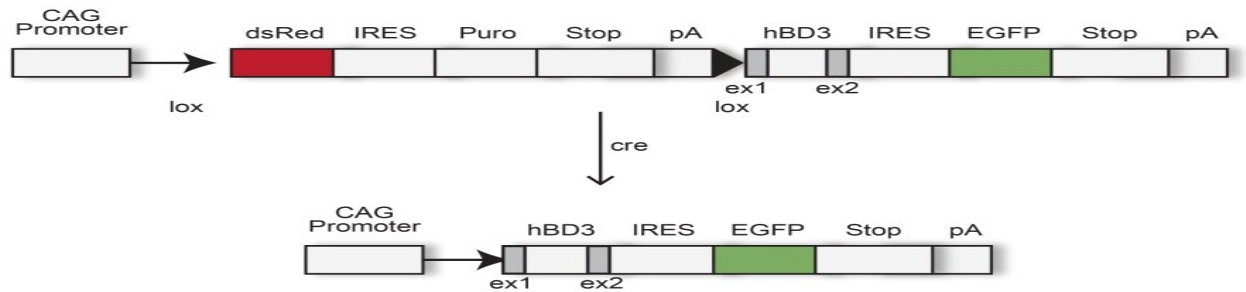

FigS1B.

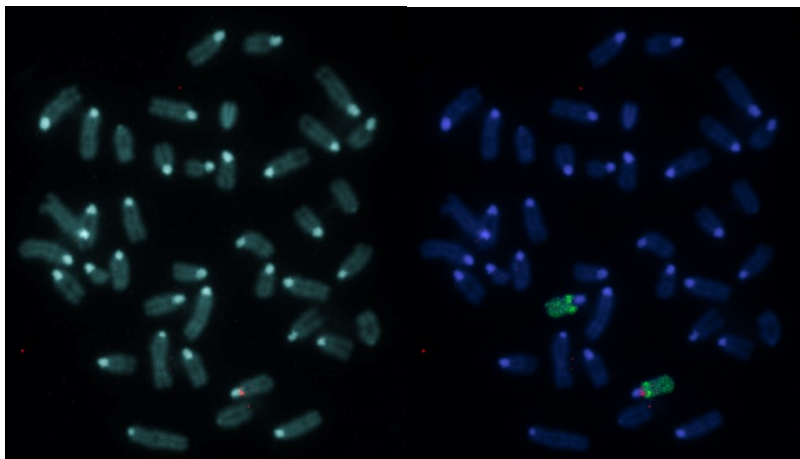

FISH with TxRd Labelled Plasmid

Mouse chromosome 12 paint

FigS1C.

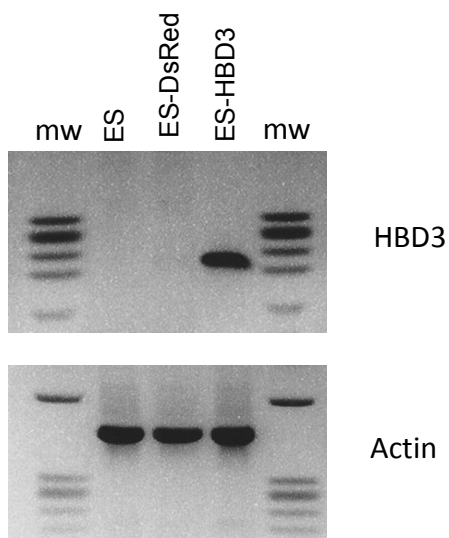

**Fig S1D**

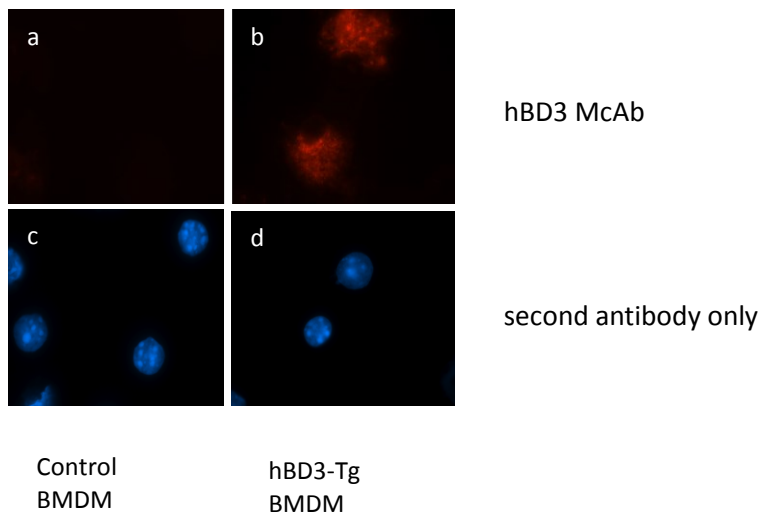

Supplement: S1 Fig — A: Transgene vector diagram: Control and hBD3 expressing vectors were created from pTLC vector (kind gift of Josh Brickman). CAG indicate the CAAG promoter (cytomeglavirus enhancer and chicken β-actin) and the arrow indicates direction of transcription; dsRed indicates the DsRed gene [58]; IRES indicates internal ribosome entry site; Puro indicates puromycin antibiotic resistance gene STOP is the translation termination signal and pA indicates polyadenylation signal; hBD3 is DEFB103 genomic fragment with exons 1 and 2 indicated by ex1 and ex2; EGFP is Enhanced Green Fluorescent Protein. ES cells (E14(iv), that were 129/Ola derived), were transfected by electroporation with ScaI linearised vector as shown in the upper region of the vector S1A Fig. Cells expressing DsRed by FACS that were shown to have normal chromosome number of n = 40, were injected into 3.5d blastocysts from C57Bl/6J mice, to create chimaeric animals. The chimaeras were bred with C57Bl/6N mice to establish heterozygotes. Homozygous mice were established at 7 backcross generations by het x het crosses and homozygotes were identified by strong DsRed fluorescence evident using a hand held lamp with a light source excitation of 440–460 nm. Homozygotes were validated by breeding i.e. all offspring carried the transgene when mated with wildtype. The ES clone used to make DsRed-Tgs was treated transiently with cre recombinase by transfection of a cre expression plasmid (kind gift of Prof. Austin Smith). These cells now no longer expressed DsRed by FACS but were EGFP positive. B: The site of transgene integration: of the vector was identified by fluorescent in situ hybridisation (FISH) and shown by chromosome painting of targeted ES cells to be on chromosome 12. C: hBD3 expression in ES cells with cre treated vector: shows that following CRE treatment the ES cells express transcript from DEFB103 (hBD3). Mw indicates Øx174 DNA cut with HaeIII and run on 3% agarose gel. DEFB103 RT-PCR from exon 1 to 2 produc [file pgen.1005673.s001.pdf]

**Figure S2:**

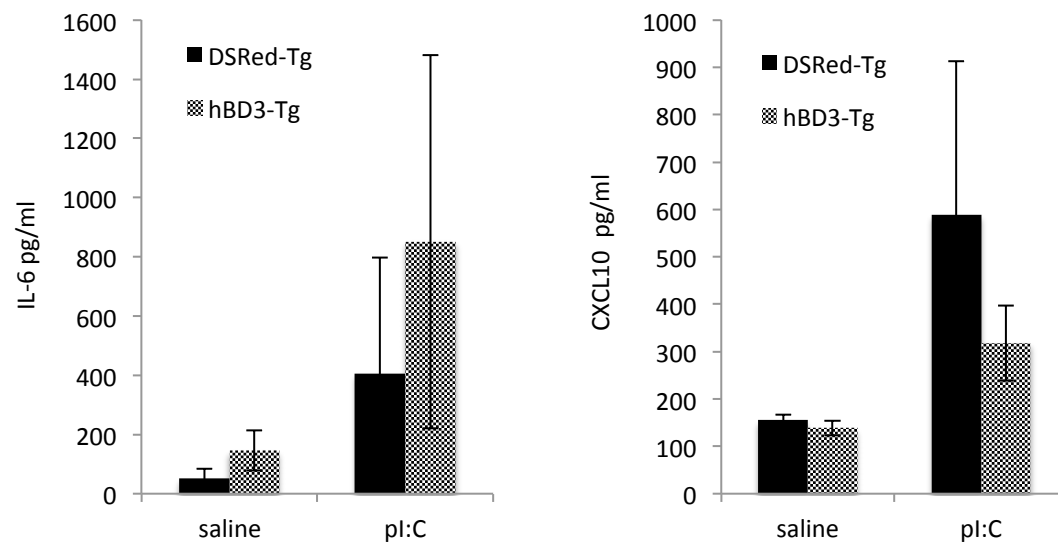

Supplement: S2 Fig — hBD3-Tg and DSRed-Tg control mice were injected i.p. with pI:C (100μg/mouse), after 4hr serum cytokine levels were measured by ELISA. (n = 6 separate mice for each treatment group). In pI:C treated hBD3-Tg mice there is a trend towards enhanced pI:C induction and reduced CXCL10 induction, compared to controls, however these data did not reach significance. (PDF) [file pgen.1005673.s002.pdf]

Figure S4

A.

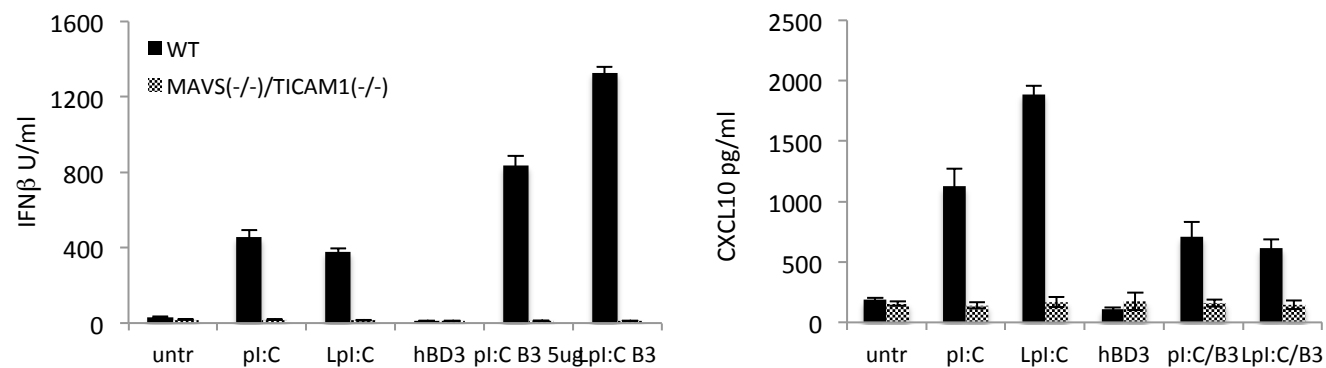

B.

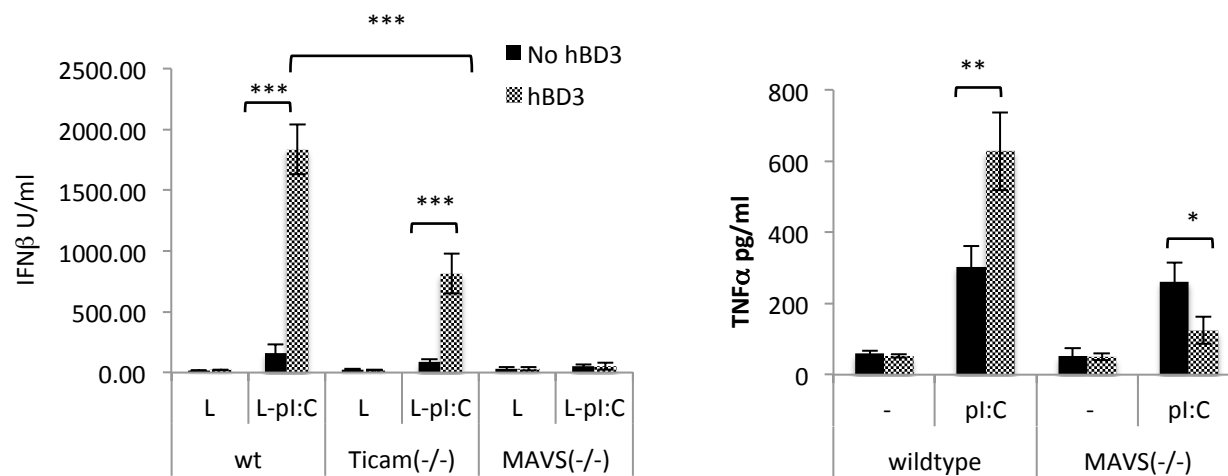

Supplement: S4 Fig — Response of BMDM from (A) Mavs(-/-)/Ticam(-/-) double knockout mice, and (B) Mavs (-/-) or Ticam(-/-) single KO BMDM were treated for 18hr with polyI:C (10μg/ml) in the presence and absence of hBD3 (5μg/ml), with or without lipofectamine (L) as indicated (L-p:C is lipofectamine with polyIC). IFNβ, CXCL10 and TNFα in cell supernatants were measured by ELISA, *p<0.05, **p<0.01, ***p<0.005 student t-test. (PDF) [file pgen.1005673.s004.pdf]

**Figure S5:**

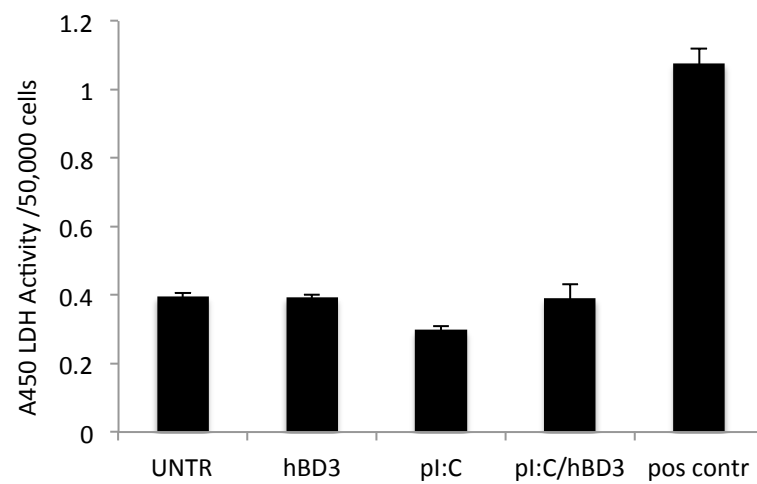

Supplement: S5 Fig — BMDM from wt mice were treated for 18hr with polyI:C (pIC) (10μg/ml) in the presence and absence of hBD3 (5μg/ml). 5x10^4^ cells were exposed to each treatment in triplicate then NADH levels measured using an LDH-Cytotoxicity Colorimetric Assay (BioVision). (PDF) [file pgen.1005673.s005.pdf]

Figure S6

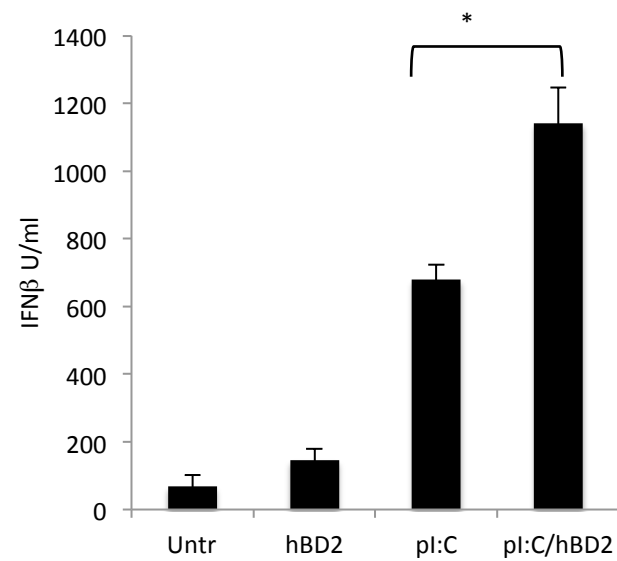

Supplement: S6 Fig — BMDM from wild type mice were treated for 18hr with polyI:C (10μg/ml) in the presence and absence of hBD2 (5μg/ml). IFNβ in cell supernatants was measured by ELISA, *p<0.01, student t-test. (PDF) [file pgen.1005673.s006.pdf]
