# Supplementary material for: Human β-D-3 Exacerbates MDA5 but Suppresses TLR3 Responses to the Viral Molecular Pattern Mimic Polyinosinic:Polycytidylic Acid
Source: PLoS Genet. 2015 Dec 8;11(12):e1005673. doi: 10.1371/journal.pgen.1005673 (PMC4672878; doi:10.1371/journal.pgen.1005673)
Supplement: S3 Fig — Images showing EEA-1 pseudocoloured red or green as indicated and no other fluorescence revealed. Fig 5Eviii and 5Evi shown again for scale and indication of merged images with the pseudocoloured EEA-1. merged Scale bar 10 microns. (PDF) [file pgen.1005673.s003.pdf]

Figure S3:

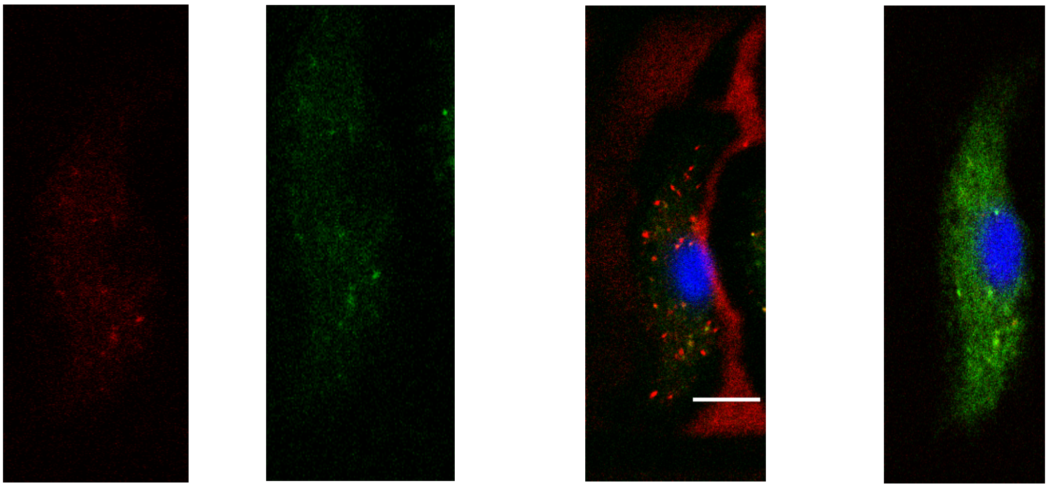

EEA-1 pseudocoloured red

EEA-1 pseudocoloured green

EEA-1 pseudocoloured green; FITC-polyIC invisible; TAMRA-hBD3 red

EEA-1 pseudocoloured red
FITC-polyIC green
TAMRA-hBD3 invisible
